# The Dentists—Kentucky State Dental Association—How It Was Organized, by Whom, and for What Purpose

**Published:** 1872-01

**Authors:** 


### THE DENTISTS—KENTUCKY STATE DENTAL ASSOCIATION—HOW IT WAS ORGANIZED, BY WHOM, AND FOR WHAT PURPOSE.

In April, 1865, a call was issued by some of our most prominent dentists for a meeting of the profession in this city, for the purpose of organizing a Dental Association. The Association was in pursuance of the call organized, composed of members of the profession of Kentucky, Tennessee, Mississippi, Indiana, Ohio, Illinois and Missouri, and was called the Central States Dental Association.

This Association having accomplished its work (i. e.) of stimulating the organization of other associations in a portion of these States, it was thought best to discontinue it. In the meantime the old Kentucky Society died out. Then, in order to revive it, and to found the new one on a more permanent basis, and in view of the failure of the Legislature to pass the law asked for, a charter of the State Association was asked of the Legislature, and was granted, whereby the following persons, viz.: Wm. M. Rogers, D. D. S.; W. G. Redman, D. D. S.; W. H. Shadoan, D. D. S.; S. Driggs, D. D. S.; F. Peabody, D. D. S.; W. H. Goddard, D. D. S.; A. S. Talbert, D. D. S.; S. Griffith, D. D. S., and G. S. Jones, D. D. S., and their successors, were created a body corporate and politic, to have perpetual succession, with the privilege of holding property to the value of ten thousand dollars ($10,000).

By the power thus granted in the charter, a majority of the incorporators met at the rooms of Drs. Goddard and Peabody, and organized by the election of officers to serve one year, or until their successors are elected.

Since the organization they have adopted a constitution and rule of order for their government, under which the Association is now working.

There have been added to the first named members the following: C. E. Dunn, D. D S.; J. W. Grant, D. D. S; J.

F. Canine, D. D. S., and G. W. Priest, D. D. S. Drs. Talbert and Griffith declined taking an active part in the Association, and were dropped. It will be seen that none but graduates in dental science are as yet members.

The object of the Association is to stimulate the profession in the State to a higher order of qualification, to create a more brotherly feeling in the profession, and to advance the interest of the profession generally.

The rules adopted for the admission of members in the Association are, first: Graduates of any regular dental college, who are in good standing in the profession, are admitted without an examination. Those who are not graduates are required to undergo an examination by the Board of Censors, who recommend them to the President, whose duty it is to issue to such as pass a satisfactory examination, a diploma or certificate of qualification, which admits the holder to membership, and to all the privileges and immunities of the profession in the State. This diploma does not confer upon the candidate the degree of D. D. S. (Doctor of Dental Surgery), but simply serves as an evidence of qualification, and protects the holder against all laws that may hereafter be enacted. The diploma is granted to all members, whether they are graduates in dental surgery or not.

It is the aim of the Association to collect a library of valuable works on dentistry and the collateral branches, to procure a microscope of high powers, and to supply it with a large collection of anatomical and dental specimens, and to provide a cabinet for the benefit of the members.

The aim of the members is to make this one of the best societies in existence. They are working quietly but surely, and in a short time we will see one of the best and most prosperous institutions of the kind in the country.

The time of meetings is annually, on the first Tuesday in June, and continue three days. In addition to that, they have adopted a system of semi-monthly meetings, which are held on he first and third Tuesday nights, at the rooms of the Louisville Dental Infirmary, No. 16 McDowell's block, corner of Fourth and Green streets. The object in holding these monthly meetings is to afford an opportunity to the members throughout the State of visiting and being at the meetings more frequently than they otherwise would have.

In connection with the Association, they have established an infirmary, for the double purpose of affording dental services to the worthy poor, and to furnish an opportunity to the members of witnessing each other's operations, thereby enabling them to give to their patients the combined skill of the profession—for such it is. These operations are of incalculable benefit in many instances.

We hope to see this Dental Association prosper. The object of the Association is not to accumulate large numbers of members, but to get good men—men who will be of benefit to the Association, and not a reproach.

---

A New Iodine Paint.—Dr. J. Warring Curran recommends (*Medical Press and Circular*, August 3, 1870,) the following paint as as an excellent application in cases of glandular enlargement and scrofulous diseases, wherein iodine is called into requisition. Rub down half an ounce of iodine and a like quantity of iodide of ammonium in a Wedgewood mortar, and gradually dissolve it in twenty ounces of rectified spirit; to this add four ounces of glycerine, shaking well the solution. Iodide of ammonium, the author thinks, is a more powerful absorbent than iodide of potassium.—*Medical News and Library*.

